# Initial Alignment for SINS Based on Pseudo-Earth Frame in Polar Regions

**DOI:** 10.3390/s17061416

**Published:** 2017-06-16

**Authors:** Yanbin Gao, Meng Liu, Guangchun Li, Xingxing Guang

**Affiliations:** College of Automation, Harbin Engineering University, Harbin 150001, China; gaoyanbin@hrbeu.edu.cn (Y.G.); lgc_67@hrbeu.edu.cn (G.L.); guangxingxing1008@163.com (X.G.)

**Keywords:** polar alignment, pseudo-Earth frame, backward process, global inertial navigation system, nonlinear filtering model

## Abstract

An accurate initial alignment must be required for inertial navigation system (INS). The performance of initial alignment directly affects the following navigation accuracy. However, the rapid convergence of meridians and the small horizontal component of rotation of Earth make the traditional alignment methods ineffective in polar regions. In this paper, from the perspective of global inertial navigation, a novel alignment algorithm based on pseudo-Earth frame and backward process is proposed to implement the initial alignment in polar regions. Considering that an accurate coarse alignment of azimuth is difficult to obtain in polar regions, the dynamic error modeling with large azimuth misalignment angle is designed. At the end of alignment phase, the strapdown attitude matrix relative to local geographic frame is obtained without influence of position errors and cumbersome computation. As a result, it would be more convenient to access the following polar navigation system. Then, it is also expected to unify the polar alignment algorithm as much as possible, thereby further unifying the form of external reference information. Finally, semi-physical static simulation and in-motion tests with large azimuth misalignment angle assisted by unscented Kalman filter (UKF) validate the effectiveness of the proposed method.

## 1. Introduction

With the development of worldwide transportation, the importance of polar navigation is increasing. The polar navigation technology can ensure the safety and reliability of vehicles when they sail in polar regions. However, due to the special geographical and electromagnetic characteristics [1], common navigation methods such as satellite navigation, radio navigation and geomagnetic navigation do not always work efficiently in polar regions [2,3]. As a result, inertial navigation system (INS), which is a highly autonomous and self-contained navigation system, becomes an optimal choice for polar navigation [4,5].

However, in polar regions, the rapid convergence of meridians makes the traditional north-oriented mechanization ineffective because the characteristic that the y-axis always points toward the north would result in both mechanical and mathematical difficulties in a platform instrument or strapdown attitude matrix [6,7]. As a result, the wander navigation system [8], grid navigation system [9], and transverse navigation system [10] have been designed and developed to solve the problem. Nonetheless, any one of these navigation systems does not have the ability of global navigation. Consequently, a combination of them is usually applied to achieve the global navigation [11]. To benefit from the understandability and familiarity, the local geographic frame is being now widely used as the navigation frame in mid-low latitude regions. As a result, the combination of the north-oriented navigation system and a wander navigation system, or a grid navigation system, or a transverse navigation system is currently selected to achieve the global navigation. Then, a need to transform the navigation parameters relative to local geographic frame to other navigation frames has also developed for global navigation. Therefore, if the initial navigation parameters relative to local geographic frame are obtained at the end of polar alignment phase, it would be more convenient to access the following polar navigation phase without any additional workloads and complexities.

Since strapdown INS (SINS) is a dead-reckoning navigation system, an accurate initial alignment is required before navigation [12]. The performance of initial alignment directly affects the following navigation accuracy. For SINS, since initial velocity and position can be easily obtained by external reference navigation systems such as GPS, the purpose of initial alignment is to determine the coordinate transformation matrix from the body frame to computational navigation frame [13,14]. In the last few decades, an amount of work has been done for initial alignment of SINS. However, most of the previous research works are focused on the initial alignment of mid-low latitude. The polar alignment is still a severe challenge for global navigation of SINS. 

For initial alignment operated in the polar regions, two major problems need to be addressed. Firstly, the navigation errors with the traditional north-oriented frame are enlarged sharply [8]. In traditional north-oriented mechanization, the propagation of azimuth error is proportional to both linear movement error and tangent function of latitude. As a result, the attitude reference relative to local geographic frame would lose its effectiveness for polar alignment. Therefore, a suitable attitude reference frame needs to be selected for polar alignment. Secondly, the horizontal component of rotation of Earth decreases to zero gradually with the increase of latitude [15]. The degree of observability of azimuth would become smaller [16]. As a result, the initial alignment with a horizontal frame would be not achieved by self-alignment in and near the North/South Pole. Moreover, it is also difficult to obtain an accurate coarse alignment of azimuth in polar regions. Therefore, a kind of valid external observation information of filter needs to be selected to achieve polar alignment. In addition, the alignment modeling with large azimuth misalignment angle should also be designed.

For selection of attitude reference frame in polar regions, the wander frame, the grid frame, and the transverse frame have been proposed for polar navigation. However, the wander attitude reference frame just can be applied in most of polar regions because the definition of wander angle is invalid at 90-degree latitude [17]. In the grid frame [18], the grid reference line is set for determining the azimuth angle. The grid attitude reference frame is introduced for polar alignment. In the transverse frame [19], a transverse attitude reference frame is defined there. According to above description, either the grid frame or the transverse frame can be selected as attitude reference frame for polar alignment [18,19,20,21]. However, just the attitude matrix relative to the grid frame or transverse frame is usually acquired when the initial alignment with these two attitude reference frames is completed. As a result, some additional transformation may need to be added to access the following polar navigation phase. Because the wander navigation system, or grid navigation system, or transverse navigation system may be selected as the following navigation system. Moreover, the transformation of attitude matrix with different reference frames would be inaccurate due to the position errors. The influence of position errors on the alignment accuracy is proportional to both the latitude error and secant function of latitude [22]. Consequently, near the pole regions, the influence would become very large. Thus, the influence of position errors on transformation accuracy must be considered in polar regions. Furthermore, the transformation of attitude matrix is also difficult due to the cumbersome derivation and computational complexity [17,18,19,20,21,22].

On the other hand, for polar alignment, the external observation information of filter also needs to be selected validly. Due to the small horizontal component of rotation of Earth, some observation information of filter applied usually in mid-low latitude would be invalid in polar regions, especially in and near pole. For initial alignment, the external position or velocity information is usually selected as the observation information of filter [23,24]. However, more time would be required to achieve the initial alignment when the external position information is selected as the observation information. The accelerated maneuver might also need to be conducted for polar alignment due to the smaller degree of observability of azimuth [25]. Hence, the external velocity reference is preferred selection for the observation information of filter. For velocity reference information, there are usually two forms: velocity information projected in body frame ($V^{b}$) and velocity information projected in the navigation frame ($V^{n}$). However, polar alignment assisted by $V^{b}$ would also be difficult to achieve due to the smaller horizontal component of rotation of Earth. As a result, $V^{n}$ should be selected as the observation information of filter to achieve the polar alignment [19,21]. This means that polar alignment with the grid frame or transverse frame needs different forms of velocity reference information due to the fact that the different attitude reference frames are employed in the grid alignment or transverse alignment. As a result, the grid alignment or transverse alignment would be disadvantageous to achieve a unified polar alignment algorithm and to unify the form of external reference information further.

According to above analysis and considering the features of global navigation of SINS, a polar alignment algorithm, which can directly obtain the attitude matrix relative to local geographic frame when the initial alignment is finished, is expected to be proposed. The transformation of attitude matrix is not influenced by position errors. Moreover, a unified polar alignment algorithm is also expected to be explored as much as possible. Then the form of external observation information of filter can also be unified for polar alignment in future. Furthermore, it would also be advantage to implement the acquirement of the observation information in a form from the external navigation device. On the other hand, since the definition of coordinate system just like that in the traditional Earth frame, the transverse frame is more immediate and convenient [19]. Therefore, an improved SINS mechanization based on pseudo-Earth frame [26], which is a generalized transverse Earth frame, is proposed in this paper. Then, a novel alignment algorithm based on pseudo-Earth frame and backward process is proposed to achieve the initial alignment in polar regions.

The purpose of this research is expected to provide a new idea to solve the problem of polar alignment and explores a unified polar alignment algorithm as much as possible. The rest of this paper is organized as follows: the proposed polar alignment scheme based on pseudo-Earth frame and backward process is presented in Section 2. In Section 3, the dynamic error modeling with large azimuth misalignment angle is designed. The nonlinear model of polar alignment is given in Section 4. The semi-physical static simulation and in-motion tests with the proposed method are presented in Section 5, and Section 6 is the conclusion.

## 2. The Polar Alignment Scheme Based on Pseudo-Earth Frame

### 2.1. The Mechanization of SINS with Pseudo-Earth Frame

**Figure 1:**
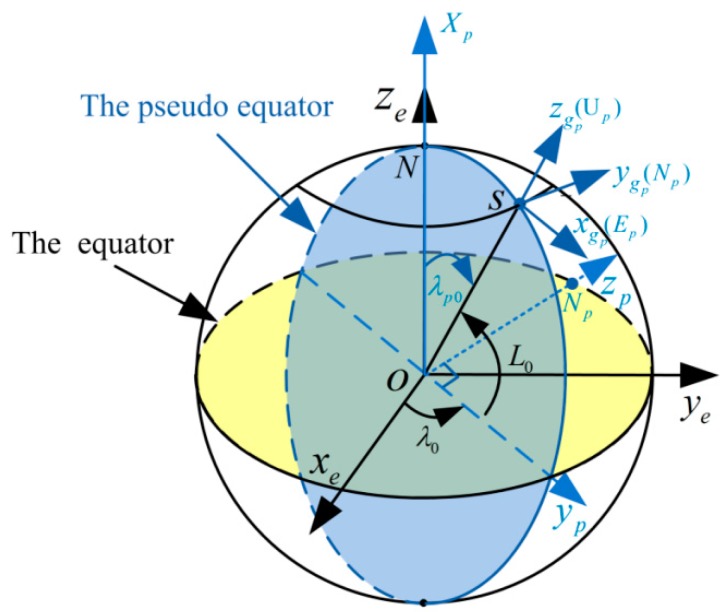
Thepseudo coordinate system.

The pseudo-Earth frame (p-frame) can be obtained by revolving Earth-Centered Earth-Fixed frame (e-frame). As shown in Figure 1, the origin of the p-frame ($o_{p}x_{p}y_{p}z_{p}$) is still at the Earth’s mass center; $N_{p}$ denotes the pseudo-North Pole; the $o_{p}x_{p}$ axis overlaps with previous $o_{e}z_{e}$ axis; the $o_{p}y_{p}$ axis is pointing from the center of Earth to the projection point of initial position of vehicle S on the equatorial plane; and the $o_{p}z_{p}$ axis is perpendicular to the $o_{p}y_{p}$ axis and conforms to the right hand rule, while laying in the equatorial plane. The detailed definition of the p-frame can be found in [26]. Supposing the initial longitude, latitude and altitude of vehicle are $\lambda_{0}$, $L_{0}$ and $h_{0}$, respectively. Then, the rotating matrix between the p-frame and e-frame can be written as:

(1) $$C_{e}^{p}=C_{x}(\lambda_{0}-90^{\circ })\cdot C_{y}(-90^{\circ })$$

where $C_{x}(\lambda_{0}-90^{\circ })=\left[\begin{array}{ccc} 1 & 0 & 0\\ 0 & \cos(\lambda_{0}-90^{\circ }) & \sin(\lambda_{0}-90^{\circ })\\ 0 & -\sin(\lambda_{0}-90^{\circ }) & \cos(\lambda_{0}-90^{\circ }) \end{array}\right]$; $C_{y}(-90^{\circ })=\left[\begin{array}{ccc} \cos(-90^{\circ }) & 0 & -\sin(-90^{\circ })\\ 0 & 1 & 0\\ \sin(-90^{\circ }) & 0 & \cos(-90^{\circ }) \end{array}\right]$.

For the definitions of pseudo-Earth coordinate system, they are similar to the ones in traditional Earth coordinate system [26]. The position of vehicle is determined by pseudo longitude $\lambda_{p}$, pseudo latitude $L_{p}$, and pseudo altitude $h_{p}$. The pseudo geographic frame $o_{g_{p}}x_{g_{p}}y_{g_{p}}z_{g_{p}}$ (namely, $E_{p}N_{p}U_{p}$) is also similar to the East–North–Up geographic frame (namely, g-frame), which is shown in Figure 1. The initial pseudo position of vehicle can also be given by:

(2) $${\left[\begin{array}{ccc} L_{p0} & \lambda_{p0} & h_{p0} \end{array}\right]}^{T}={\left[\begin{array}{ccc} 0^{\circ } & 90^{\circ }-L_{0} & h_{0} \end{array}\right]}^{T}$$

According to above definitions, the pseudo-Earth frame is a generalized transverse Earth frame built with the initial position of vehicle in fact [17]. Therefore, the pseudo navigation mechanization of SINS is also similar to the one with transversal Earth coordinate system. In pseudo navigation mechanization, the $g_{p}$-frame is selected as navigation frame (n-frame). Only the angular velocity of p-frame relative to inertial frame (i-frame) differs from that in the local-level coordinate system. As a result, the pseudo navigation equations can be written by [17,26]:

(3) $${\dot{C}}_{b}^{n}=C_{b}^{n}(\omega_{nb}^{b}\times )$$

(4) $${\dot{V}}_{p}^{n}=C_{b}^{n}f^{b}-(2\omega_{ip}^{n}+\omega_{pn}^{n})\times V_{p}^{n}+g^{n}$$

(5a) $${\dot{L}}_{p}=\frac{V_{N_{p}}}{R+h_{p}}$$

(5b) $${\dot{\lambda }}_{p}=\frac{V_{E_{p}}\sec L_{p}}{R+h_{p}}$$

(5c) $${\dot{h}}_{p}=V_{U_{p}}$$

where (∗×) denotes the skew symmetric matrix of (∗); $\omega_{ab}^{c}$ denotes the rotation angular rate of frame b relative to frame a represented in the c frame; $\omega_{ip}^{n}=[-\Omega \sin\lambda_{p}-\Omega \sin L_{p}\cos\lambda_{p}$ ${\Omega \cos L_{p}\cos\lambda_{p}]}^{T}$; Ω denotes the angular rate of Earth rotation; $\omega_{pn}^{n}=[\begin{array}{cc} -V_{N_{p}}/\left(R+h_{p}\right) & V_{E_{p}}/\left(R+h_{p}\right) \end{array}$ $\omega_{pn}^{n}={V_{E_{p}}\tan L_{p}/\left(R+h_{p}\right)]}^{T}$; R denotes the Earth radius; $\omega_{nb}^{b}=\omega_{ib}^{b}-C_{n}^{b}\omega_{in}^{n}$, $\omega_{in}^{n}=\omega_{ip}^{n}+\omega_{pn}^{n}$, $C_{n}^{b}={\left(C_{b}^{n}\right)}^{T}$; $\omega_{ib}^{b}$ denotes the outputs of gyro; $f^{b}$ denotes the specific force; and $g^{n}$ denotes the gravity vector in the n-frame, $g^{n}\approx {\left[\begin{array}{ccc} 0 & 0 & -g \end{array}\right]}^{T}$.

Since the pseudo-Earth frame is a generalized transverse Earth frame and the pseudo latitude at the initial position (namely, $L_{p0}$) is always zero from Equation (2), the pseudo-Earth frame can solve the problem of attitude reference frame of polar alignment. In addition, the polar alignment algorithm based on pseudo-Earth frame is also easy to implement because it is similar to the polar alignment algorithm with the transverse Earth frame. However, if the strapdown attitude matrix relative to g-frame is obtained by direct transformation with both the pseudo attitude matrix and the relationship of them, the attitude matrix would be still influenced by the position errors. The cumbersome derivation and computational complexity must also be confronted. Nevertheless, by computation, it is easily find that the initial transformation matrix between $g_{p}$-frame and g-frame at initial position is a constant matrix and never change with the initial position of vehicle. As a result, it would be very advantageous to achieve the transformation of attitude matrix between g-frame and $g_{p}$-frame without the influence of position errors and the cumbersome computation. The initial transformation matrix $C_{g}^{g_{p}}(0)$ can also be given by:

(6) $$C_{g}^{g_{p}}(0)=C_{p}^{g_{p}}(0)\cdot C_{e}^{p}\cdot C_{g}^{e}(0)$$

where $C_{p}^{g_{p}}(0)=\left[\begin{array}{ccc} -\sin\lambda_{p0} & \cos\lambda_{p0} & 0\\ -\sin L_{p0}\cos\lambda_{p0} & -\sin L_{p0}\sin\lambda_{p0} & \cos L_{p0}\\ \cos L_{p0}\cos\lambda_{p0} & \cos L_{p0}\sin\lambda_{p0} & \sin L_{p0} \end{array}\right]$; $C_{g}^{e}(0)=\left[\begin{array}{ccc} -\sin\lambda_{0} & -\sin L_{0}\cos\lambda_{0} & \cos L_{0}\cos\lambda_{0}\\ \cos\lambda_{0} & -\sin L_{0}\sin\lambda_{0} & \cos L_{0}\sin\lambda_{0}\\ 0 & \cos L_{0} & \sin L_{0} \end{array}\right]$.

Substituting Equations (1) and (2) into Equation (6), the initial transformation matrix $C_{g}^{g_{p}}(0)$ can be rewritten by:

(7) $$C_{g}^{g_{p}}(0)=\left[\begin{array}{ccc} 0 & -1 & 0\\ 1 & 0 & 0\\ 0 & 0 & 1 \end{array}\right]$$

From Equation (7), we can obtain that the $g_{p}$-frame is identical to the South–East–Up geographic frame at initial position. As a result, if the pseudo attitude matrix of vehicle at initial position is obtained when the initial alignment is completed, it would be equal to the attitude matrix relative to local geographic frame is obtained without influence of transformation errors and cumbersome computation. Moreover, the coarse alignment method needs also not to be redesigned, and the traditional coarse alignment algorithm can be applied directly. Therefore, the pseudo navigation mechanization would be simpler to solve the problem of attitude reference frame for polar alignment. Nevertheless, the pseudo attitude matrix of vehicle at initial position is expected to be obtained when the initial alignment based on pseudo navigation mechanization is completed.

**Remark.**  *If the initial latitude of vehicle relative to* e*-frame is 90°, the initial longitude of vehicle can choose the arbitrary value with* $\left[-180^{\circ },\;180^{\circ }\right]$*. Then, the pseudo-Earth frame is built based on the selected longitude. The initial azimuth angle can still be obtained by the initial pseudo azimuth angle from Equation (7). Nevertheless, we should notice that the azimuth angle relative to* g*-frame in pole is the angle between the vehicle direction and the selected longitude.*

### 2.2. Backward Process for Pseudo Navigation System

With electronic technology development, the capabilities of the navigation computer become large. The backward process of data has been proposed for initial alignment [27,28,29]. By utilizing the saved data of inertial measurement unit (IMU) with the opposite time sequence, the backward process can accelerate the process of alignment. Nevertheless, here the requirement that the pseudo attitude matrix of vehicle at initial position is obtained at the end of alignment phase is expected to be realized by utilizing the saved IMU data. If the pseudo starpdown attitude matrix and the pseudo position in backward process are identical to the forward ones at the same time, the pseudo position with backward process can return to initial position. Then, the initial attitude matrix relative to g-frame can be obtained from Equation (7). On the other hand, the backward process can still be applied to decrease the alignment time. 

From Equations (3)–(5), the discrete form of forward pseudo navigation equations can be given as follows:

(8) $$C_{bk}^{n}=C_{bk-1}^{n}(I+T\omega_{nbk-1}^{b}\times )$$

(9) $$V_{pk}^{n}=V_{pk-1}^{n}+TC_{bk-1}^{n}f_{k-1}^{b}-T(2\omega_{ipk-1}^{n}+\omega_{pnk-1}^{n})\times V_{pk-1}^{n}+Tg_{k-1}^{n}$$

(10a) $$L_{pk}=L_{pk-1}+T\frac{V_{N_{p}k-1}}{R+h_{pk-1}}$$

(10b) $$\lambda_{pk}=\lambda_{pk-1}+T\frac{V_{E_{p}k-1}\sec L_{pk-1}}{R+h_{pk-1}}$$

(10c) $$h_{pk}=h_{pk-1}+TV_{U_{p}k-1}$$

where k is the discrete time index for forward computation, and k=0,1,2,⋯m; $A_{k}$ denotes the computed value of A at time $t_{k}$, while $A_{k-1}$ denotes the computed value of A at time $t_{k-1}$; T denotes the sampling period; I denotes the identity matrix. 

Then, through further transposition, the backward pseudo navigation equations can also be obtained:

(11) $$C_{bk-1}^{n}=C_{bk}^{n}{\left(I+T\omega_{nbk-1}^{b}\times \right)}^{-1}\approx C_{bk}^{n}(I-T\omega_{nbk-1}^{b}\times )$$

(12) $$V_{pk-1}^{n}=V_{pk}^{n}-TC_{bk-1}^{n}f_{k-1}^{b}+T(2\omega_{ipk-1}^{n}+\omega_{pnk-1}^{n})\times V_{pk-1}^{n}-Tg_{k-1}^{n}$$

(13a) $$L_{pk-1}=L_{pk}-T\frac{V_{N_{p}k-1}}{R+h_{pk-1}}$$

(13b) $$\lambda_{pk-1}=\lambda_{pk}-T\frac{V_{E_{p}k-1}\sec L_{pk-1}}{R+h_{pk-1}}$$

(13c) $$h_{pk-1}=h_{pk}-TV_{U_{p}k-1}$$

Moreover, assuming the discrete time index for backward computation is l(l=0,1,2,⋯m), the backward pseudo navigation equations can be written as follows:

(14) $${\overset{\leftarrow }{C}}_{bl}^{n}={\overset{\leftarrow }{C}}_{bl-1}^{n}(I+T{\overset{\leftarrow }{\omega }}_{nbl-1}^{b}\times )$$

(15) $${\overset{\leftarrow }{V}}_{pl}^{n}={\overset{\leftarrow }{V}}_{pl-1}^{n}+T{\overset{\leftarrow }{C}}_{bl-1}^{n}{\overset{\leftarrow }{f}}_{l-1}^{b}-T(2{\overset{\leftarrow }{\omega }}_{ipl-1}^{n}+{\overset{\leftarrow }{\omega }}_{pnl-1}^{n})\times {\overset{\leftarrow }{V}}_{pl-1}^{n}+T\;{\overset{\leftarrow }{g}}_{l-1}^{n}$$

(16a) $${\overset{\leftarrow }{L}}_{pl}={\overset{\leftarrow }{L}}_{pl-1}+T\frac{{\overset{\leftarrow }{V}}_{N_{p}l-1}}{R+{\overset{\leftarrow }{h}}_{pl-1}}$$

(16b) $${\overset{\leftarrow }{\lambda }}_{pl}={\overset{\leftarrow }{\lambda }}_{pl-1}+T\frac{{\overset{\leftarrow }{V}}_{E_{p}l-1}\sec{\overset{\leftarrow }{L}}_{pl-1}}{R+{\overset{\leftarrow }{h}}_{pl-1}}$$

(16c) $${\overset{\leftarrow }{h}}_{pl}={\overset{\leftarrow }{h}}_{pl-1}+T{\overset{\leftarrow }{V}}_{U_{p}l-1}$$

where the superscript “←” denotes this term is for the backward process.

**Figure 2:**
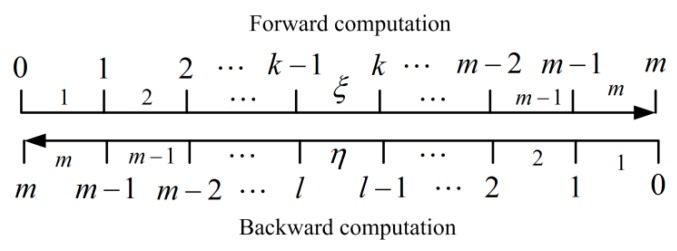
The corresponding relations of time index between forward process and backward process.

Defining ξ denotes the ξ th sample with the normal time sequence (ξ=1,2,⋯m) and η denotes the η th sample with the opposite time sequence (η=1,2,⋯m). According to the forward computed process and backward computed process above, the corresponding relations of time index can be shown in Figure 2.

According to the analysis above, the backward strapdown attitude matrix should equal to the forward one at the same time. As a result, the corresponding terms of Equations (11) and (14) should also be equal. Comparing Equation (11) and Equation (14), we can obtain:

(17) $$\left\{ \begin{array}{l} {\overset{\leftarrow }{C}}_{bl}^{n}=C_{bk-1}^{n}\\ {\overset{\leftarrow }{C}}_{bl-1}^{n}=C_{bk}^{n} \end{array}\right.$$

(18) $$T{\overset{\leftarrow }{\omega }}_{nbl-1}^{b}=-T\omega_{nbk-1}^{b}$$

Considering the corresponding relations of time index from Figure 2, we further have:

(19) l+k−1=m

(20) $${\overset{\leftarrow }{\omega }}_{nb}^{b}(\eta )=-\omega_{nb}^{b}(\xi )$$

Obviously, ξ=k, η=l. Combining Equations (19) and (20) can be rewritten by:

(21) $${\overset{\leftarrow }{\omega }}_{nb}^{b}(l)=-\omega_{nb}^{b}(m-l+1)$$

On the other hand, it is also expected that the backward pseudo position could be identical to the forward ones at the same time. Thus, comparing the corresponding terms of Equations (13) and (16), we can similarly obtain:

(22) $$\left\{ \begin{array}{l} l+k-1=m\\ {\overset{\leftarrow }{V}}_{E_{p}}(\eta )=-V_{E_{p}}(\xi )\\ {\overset{\leftarrow }{V}}_{N_{p}}(\eta )=-V_{N_{p}}(\xi )\\ {\overset{\leftarrow }{V}}_{U_{p}}(\eta )=-V_{U_{p}}(\xi ) \end{array}\right.$$

Then, we can further have:

(23) $${\overset{\leftarrow }{V}}_{p}^{n}(l)=-V_{p}^{n}(m-l+1)$$

From Equation (23), we can conclude that the sign of the pseudo velocity in backward process should be opposite to the corresponded one in forward process. Therefore, after taking opposite, Equation (12) should equal to Equation (15), correspondingly. Then we can get:

(24) $$\left\{ \begin{array}{l} l+k-1=m\\ {\overset{\leftarrow }{f}}^{b}(\eta )=f^{b}(\xi )\\ {\overset{\leftarrow }{g}}^{n}(\eta )=g^{n}(\xi )\\ \left[2{\overset{\leftarrow }{\omega }}_{ip}^{n}(\eta )+{\overset{\leftarrow }{\omega }}_{pn}^{n}(\eta )]\times {\overset{\leftarrow }{V}}_{p}^{n}(\eta )=[2\omega_{ip}^{n}(\xi )+\omega_{pn}^{n}(\xi )]\times V_{p}^{n}(\xi \right) \end{array}\right.$$

From Equation (24), we have:

(25) $${\overset{\leftarrow }{f}}^{b}(l)=f^{b}(m-l+1)$$

(26) $${\overset{\leftarrow }{g}}^{n}(l)=g^{n}(m-l+1)$$

(27) $$\left[2{\overset{\leftarrow }{\omega }}_{ip}^{n}(l)+{\overset{\leftarrow }{\omega }}_{pn}^{n}(l)]\times {\overset{\leftarrow }{V}}_{p}^{n}(l)=[2\omega_{ip}^{n}(m-l+1)+\omega_{pn}^{n}(m-l+1)]\times V_{p}^{n}(m-l+1\right)$$

From Equations (25) and (26), the signs of the outputs of accelerometer and the gravity in backward process should be the same as the forward ones. Moreover, substituting Equation (23) into Equation (27), we can obtain:

(28) $${\overset{\leftarrow }{\omega }}_{ip}^{n}(l)=-\omega_{ip}^{n}(m-l+1)$$

(29) $${\overset{\leftarrow }{\omega }}_{pn}^{n}(l)=-\omega_{pn}^{n}(m-l+1)$$

From Equation (28), we can conclude that the sign of the angular rate of rotation of Earth in backward process needs to be taken negative. In addition, combining with Equations (21), (28), and (29), the outputs of gyro in backward process can be obtained by:

(30) $${\overset{\leftarrow }{\omega }}_{ib}^{b}(l)=-\omega_{ib}^{b}(m-l+1)$$

From Equation (30), the sign of outputs of gyro should be opposite to the forward one in the backward process.

According to above derivation, the expectation that the pseudo starpdown attitude matrix and the pseudo position in backward process should be the same as the forward ones at the same time can be realized. While the pseudo velocity of backward process is opposite to the forward one at the same time. Moreover, the signs of the outputs of gyro and the angular rate of rotation of Earth in backward process should be opposite to the corresponded ones with the forward process. The others are all the same as the forward ones. As a result, the expectation that the pseudo attitude matrix of vehicle at initial position is obtained at the end of alignment phase can be realized by backward process with pseudo navigation mechanization. Further, the initial transformation matrix $C_{g}^{g_{p}}(0)$ can be utilized to obtain the strapdown attitude matrix relative to g-frame with a simple transformation logic and without influence of position errors.

### 2.3. The Proposed Polar Alignment Scheme with Pseudo-Earth frame

From the analysis above, the alignment algorithm based on pseudo-Earth frame and backward process can be applied to solve the problem of polar alignment. The attitude matrix relative to local geographic frame is obtained when the initial alignment is completed. Moreover, it is also easy to implement without the influence of position errors and the cumbersome computation since the initial transformation matrix is a fixed known constant matrix and never influenced by initial position of vehicle from Equation (7). 

**Figure 3:**
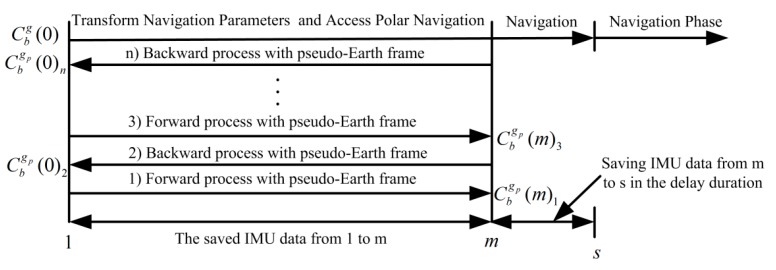
The proposed polar alignment scheme.

Supposing the IMU data are saved in first forward fine alignment and the sampling index of saved data sequence is 1 to m. Moreover, the computed pseudo attitude matrix $C_{b}^{g_{p}}{\left(m\right)}_{1}$ is also obtained at the end of first forward process. Subsequently, the backward process of the filter estimation is carried out from m to 1, and the pseudo attitude matrix $C_{b}^{g_{p}}{\left(0\right)}_{2}$ is obtained at the end of first backward process. Then, the forward process and backward process based on pseudo-Earth frame is repeatedly conducted until the initial alignment is completed. Comparing with the traditional application of the backward process, one and only difference is that the proposed alignment algorithm should end with the backward process the filter estimation from m to 1. Meanwhile, the computed pseudo attitude matrix of time 0 (namely, $C_{b}^{g_{p}}{\left(0\right)}_{n}$) is obtained. As a result, the pseudo attitude matrix at initial position is obtained by the backward process. The attitude matrix relative to g-frame is also obtained from Equation (7). Then, the navigation parameters relative to g-frame can be transformed to the corresponding reference frame of the following polar navigation system by the existing transformation program without any additional workloads and complexities. In addition, since the reciprocating process spends some time, the IMU data also need to be saved. The sampling index of saved data in the delay duration is represented from m to s. According to the transformational initial navigation parameters, the navigation calculation is conducted from the first sample to the sth sample by the selected polar navigation system, such as the wander navigation system, grid navigation system and transverse navigation system and so on. Then, the SINS accesses the navigation phase. The proposed polar alignment scheme based on pseudo-Earth frame and backward process is shown in Figure 3.

**Figure 4:**
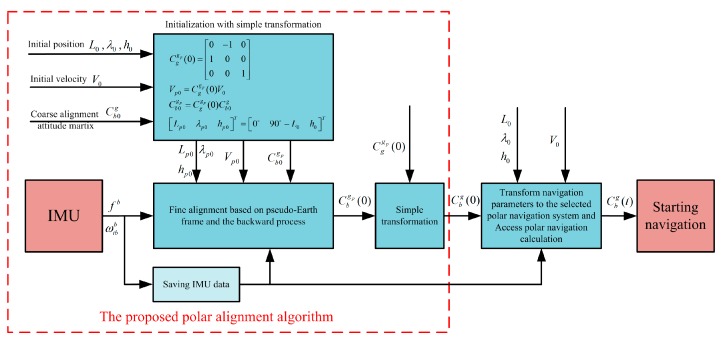
The implementation of the proposed alignment algorithm.

In addition, the implementation of the proposed alignment algorithm is also illustrated in Figure 4. As shown in Figure 4, the traditional coarse alignment algorithm can be still applied to the proposed polar alignment process. Only an initialization with simple transformation is conducted to access the next fine alignment associated with pseudo-Earth frame. Moreover, since the initial pseudo latitude is always zero, the alignment model with the pseudo-Earth frame would be always equivalent to the traditional alignment model of equatorial regions. As a result, the initial alignment with the pseudo-Earth frame can solve the attitude reference problem of polar alignment. Furthermore, since the initial transformation matrix $C_{g}^{g_{p}}(0)$ is a fixed known constant matrix, the backward process of SINS is proposed here, thereby obtaining the pseudo attitude matrix of vehicle at initial position at the end of initial alignment. As a result, the initial transformation matrix can be cleverly applied to obtain the strapdown attitude matrix relative to g-frame with a simple transformation and without influence of position errors. Thus, there is less cumbersome derivation and low computational complexity in the process of transformation, thereby decreasing the complexity of program and the burden of computer. Moreover, the backward process can still accelerate the filtering estimation and decrease the alignment time. On the other hand, the strapdown attitude matrix relative to g-frame is obtained at the end of alignment phase. It would also be more convenient to access any one of the following polar navigation systems. Because the existing transformation program of navigation parameters in global navigation system can be utilized directly, it would make it possible to achieve the unification of polar alignment algorithm. Therefore, the proposed polar alignment algorithm has superior performance.

## 3. Pseudo Navigation Error Equations with Large Azimuth Misalignment Angle

Considering that an accurate coarse alignment of azimuth is difficult to obtain in polar regions, the dynamic error modeling with large azimuth misalignment angle is designed in this section. Defining the pseudo misalignment angle between computed navigational frame ($n^{\prime }$-frame) and n-frame is $\phi_{p}={\left[\begin{array}{ccc} \phi_{xp} & \phi_{yp} & \phi_{zp} \end{array}\right]}^{T}$. Here, the pseudo-pitch and pseudo-roll misalignment angles (namely, $\phi_{xp}$ and $\phi_{yp}$) are small misalignment angles. While the pseudo-azimuth angle should be considered as a large misalignment angle. Then, the rotating matrix between $n^{\prime }$-frame and n-frame can be written as [30]:

(31) $$C_{n}^{n^{\prime }}=C_{\phi_{yp}}C_{\phi_{xp}}C_{\phi_{zp}}=\left[\begin{array}{ccc} c\phi_{yp}c\phi_{zp}-s\phi_{yp}s\phi_{xp}s\phi_{zp} & c\phi_{yp}s\phi_{zp}+s\phi_{yp}s\phi_{xp}c\phi_{zp} & -s\phi_{yp}c\phi_{xp}\\ -c\phi_{xp}s\phi_{zp} & c\phi_{xp}c\phi_{zp} & s\phi_{xp}\\ s\phi_{yp}c\phi_{zp}+c\phi_{yp}s\phi_{xp}s\phi_{zp} & s\phi_{yp}s\phi_{zp}-c\phi_{yp}s\phi_{xp}c\phi_{zp} & c\phi_{yp}c\phi_{xp} \end{array}\right]$$

where $s\phi_{jp}$ and $c\phi_{jp}$ represent the sine function and cosine function, respectively, and j=x,y,z.

Defining the angular velocity of $n^{\prime }$-frame relative to n-frame is $\omega_{nn^{\prime }}^{n^{\prime }}$, and the relationship between $\omega_{nn^{\prime }}^{n^{\prime }}$ and $\phi_{p}$ can be given by [30]:

(32) $$\omega_{nn^{\prime }}^{n^{\prime }}=C_{\phi_{yp}}C_{\phi_{xp}}\left[\begin{array}{c} 0\\ 0\\ {\dot{\phi }}_{zp} \end{array}\right]+C_{\phi_{yp}}\left[\begin{array}{c} {\dot{\phi }}_{xp}\\ 0\\ 0 \end{array}\right]+\left[\begin{array}{c} 0\\ {\dot{\phi }}_{zp}\\ 0 \end{array}\right]=A\left[\begin{array}{c} {\dot{\phi }}_{xp}\\ {\dot{\phi }}_{yp}\\ {\dot{\phi }}_{zp} \end{array}\right]$$

From Equation (32), we have:

(33) $${\dot{\phi }}_{p}=A^{-1}\omega_{nn^{\prime }}^{n^{\prime }}$$

where

(34) $$A^{-1}=\frac{1}{c\phi_{xp}}\left[\begin{array}{ccc} c\phi_{yp}c\phi_{xp} & 0 & s\phi_{yp}c\phi_{xp}\\ s\phi_{yp}s\phi_{xp} & c\phi_{xp} & -c\phi_{yp}s\phi_{xp}\\ -s\phi_{yp} & 0 & c\phi_{yp} \end{array}\right]$$

Only considering that the pseudo-azimuth angle is a large misalignment angle here, Equations (31) and (34) can be rewritten by:

(35) $$C_{n}^{n^{\prime }}=\left[\begin{array}{ccc} c\phi_{zp} & s\phi_{zp} & -\phi_{yp}\\ -s\phi_{zp} & c\phi_{zp} & \phi_{xp}\\ \phi_{yp}c\phi_{zp}+\phi_{xp}s\phi_{zp} & \phi_{yp}s\phi_{zp}-\phi_{xp}c\phi_{zp} & 1 \end{array}\right]$$

(36) $$A^{-1}=\left[\begin{array}{ccc} 1 & 0 & \phi_{yp}\\ 0 & 1 & -\phi_{xp}\\ -\phi_{yp} & 0 & 1 \end{array}\right]$$

Due to the existence of misalignment angles and gyro drifts, the differential equation of actual computed pseudo strapdown attitude matrix can be written as:

(37) $${\dot{C}}_{b}^{n^{\prime }}=C_{b}^{n^{\prime }}({\tilde{\omega }}_{nb}^{b}\times )$$

where

(38) $${\tilde{\omega }}_{nb}^{b}=(\omega_{ib}^{b}+\varepsilon^{b})-C_{n^{\prime }}^{b}(\omega_{in}^{n}+\delta \omega_{in}^{n})$$

where $\varepsilon^{b}$ denotes the gyro drifts, $\delta \omega_{in}^{n}$ denotes the calculated error of $\omega_{in}^{n}$.

Since

(39) $$C_{b}^{n}=C_{n^{\prime }}^{n}C_{b}^{n^{\prime }}$$

differentiating both sides of Equation (39) gives:

(40) $${\dot{C}}_{b}^{n}={\dot{C}}_{n^{\prime }}^{n}C_{b}^{n^{\prime }}+C_{n^{\prime }}^{n}{\dot{C}}_{b}^{n^{\prime }}$$

Substituting Equations (3), (37), and ${\dot{C}}_{n^{\prime }}^{n}=C_{n^{\prime }}^{n}(\omega_{nn^{\prime }}^{n^{\prime }}\times )$ into Equation (40), we can obtain:

(41) $$C_{n^{\prime }}^{n}(\omega_{nn^{\prime }}^{n^{\prime }}\times )C_{b}^{n^{\prime }}=C_{b}^{n}(\omega_{nb}^{b}\times )-C_{n^{\prime }}^{n}C_{b}^{n^{\prime }}({\tilde{\omega }}_{nb}^{b}\times )$$

Multiplying the left by $C_{n}^{n^{\prime }}$ and the right by $C_{n^{\prime }}^{b}$, we further have:

(42) $$(\omega_{nn^{\prime }}^{n^{\prime }}\times )=C_{b}^{n^{\prime }}(\omega_{nb}^{b}\times )C_{n^{\prime }}^{b}-C_{b}^{n^{\prime }}({\tilde{\omega }}_{nb}^{b}\times )C_{n^{\prime }}^{b}$$

Then, Equation (42) can be rewritten by:

(43) $$\omega_{nn^{\prime }}^{n^{\prime }}=C_{b}^{n^{\prime }}\omega_{nb}^{b}-C_{b}^{n^{\prime }}{\tilde{\omega }}_{nb}^{b}$$

Substituting Equation (38) and $\omega_{nb}^{b}=\omega_{ib}^{b}-C_{n}^{b}\omega_{in}^{n}$ into Equation (43), we can obtain:

(44) $$\omega_{nn^{\prime }}^{n^{\prime }}=(I-C_{n}^{n^{\prime }}){\tilde{\omega }}_{in}^{n}+C_{n}^{n^{\prime }}\delta \omega_{in}^{n}-C_{b}^{n^{\prime }}\varepsilon^{b}$$

From Equation (33), the pseudo attitude error equation with large azimuth misalignment angle can be obtained by:

(45) $${\dot{\phi }}_{p}=A^{-1}\omega_{nn^{\prime }}^{n}=A^{-1}\left[(I-C_{n}^{n^{\prime }}){\tilde{\omega }}_{in}^{n}+C_{n}^{n^{\prime }}\delta \omega_{in}^{n}-C_{b}^{n^{\prime }}\varepsilon^{b}\right]$$

where

(46) $$\delta \omega_{in}^{n}=\delta \omega_{ip}^{n}+\delta \omega_{pn}^{n}$$

(47) $$\left\{ \begin{array}{l} \delta \omega_{ip}^{n}={\left[\begin{array}{ccc} \omega_{11} & \omega_{12} & \omega_{13} \end{array}\right]}^{T}\\ \omega_{11}=-\Omega \cos\lambda_{p}\delta \lambda_{p}\\ \omega_{12}=-\Omega \cos L_{p}\cos\lambda_{p}\delta L_{p}+\Omega \sin L_{p}\sin\lambda_{p}\delta \lambda_{p}\\ \omega_{13}-\Omega \sin L_{p}\cos\lambda_{\;p}\delta L_{p}-\Omega \cos L_{p}\sin\lambda_{p}\delta \lambda_{p} \end{array}\right.$$

(48) $$\left\{ \begin{array}{l} \delta \omega_{pn}^{n}={\left[\begin{array}{ccc} \omega_{21} & \omega_{22} & \omega_{23} \end{array}\right]}^{T}\\ \omega_{21}=\frac{V_{N_{p}}}{{\left(R+h_{p}\right)}^{2}}\delta h_{p}-\frac{\delta V_{N_{p}}}{R+h_{p}}\\ \omega_{22}=-\frac{V_{E_{p}}}{{\left(R+h_{p}\right)}^{2}}\delta h_{p}+\frac{\delta V_{E_{p}}}{R+h_{p}}\\ \omega_{23}=\frac{V_{E_{p}}}{R+h_{p}}\sec^{2}L_{p}\delta L_{p}-\frac{V_{E_{p}}\tan L_{p}}{{\left(R+h_{p}\right)}^{2}}\delta h_{p}+\frac{\delta V_{E_{p}}}{R+h_{p}}\tan L_{p} \end{array}\right.$$

Since the measurement errors and calculation errors exist, the differential equation of actual computed pseudo velocity can be written as:

(49) $${\dot{\tilde{V}}}_{p}^{n}=C_{b}^{n^{\prime }}{\tilde{f}}^{b}-(2{\tilde{\omega }}_{ip}^{n}+{\tilde{\omega }}_{pn}^{n})\times {\tilde{V}}_{p}^{n}+{\tilde{g}}^{n}$$

where ${\tilde{V}}_{p}^{n}=V_{p}^{n}+\delta V_{p}^{n}$, ${\tilde{f}}^{b}=f^{b}+\nabla^{b}$, ${\tilde{\omega }}_{ip}^{n}=\omega_{ip}^{n}+\delta \omega_{ip}^{n}$, ${\tilde{\omega }}_{pn}^{n}=\omega_{pn}^{n}+\delta \omega_{pn}^{n}$, ${\tilde{g}}^{n}=g^{n}+\delta g^{n}$, and $\nabla^{b}$ denotes the zero-bias of accelerometer.

Then the pseudo velocity error equation can be obtained as:

(50) $$\delta {\dot{V}}_{p}^{n}={\dot{\tilde{V}}}_{p}^{n}-{\dot{V}}_{p}^{n}$$

Ignoring $\delta g^{n}$ and second order small quantities, substituting Equations (4) and (49) into Equation (50), can obtain:

(51) $$\delta {\dot{V}}_{p}^{n}=(I-C_{n^{\prime }}^{n})C_{b}^{n^{\prime }}{\tilde{f}}^{b}-(2\delta \omega_{ip}^{n}+\delta \omega_{pn}^{n})\times {\tilde{V}}_{p}^{n}-(2{\tilde{\omega }}_{ip}^{n}+{\tilde{\omega }}_{pn}^{n})\times \delta V_{p}^{n}+C_{n^{\prime }}^{n}C_{b}^{n^{\prime }}\nabla^{b}$$

Defining ${\tilde{L}}_{p}=L_{p}+\delta L_{p}$, ${\tilde{\lambda }}_{p}=\lambda_{p}+\delta \lambda_{p}$, ${\tilde{h}}_{p}=h_{p}+\delta h_{p}$, we can obtain the pseudo position error equations from Equation (5). They are can be expressed as:

(52) $$\left\{ \begin{array}{l} \delta {\dot{L}}_{p}={\dot{\tilde{L}}}_{p}-{\dot{L}}_{p}=-\frac{{\tilde{V}}_{N_{p}}}{R+{\tilde{h}}_{p}}\delta h_{p}+\frac{\delta V_{N_{p}}}{R+{\tilde{h}}_{p}}\\ \delta {\dot{\lambda }}_{p}={\dot{\tilde{\lambda }}}_{p}-\lambda_{p}=\frac{{\tilde{V}}_{E_{p}}\tan{\tilde{L}}_{p}\sec{\tilde{L}}_{p}}{R+{\tilde{h}}_{p}}\delta L_{p}-\frac{{\tilde{V}}_{E_{p}}\sec{\tilde{L}}_{p}}{{\left(R+{\tilde{h}}_{p}\right)}^{2}}\delta h_{p}+\frac{\delta V_{E_{p}}}{R+{\tilde{h}}_{p}}\sec{\tilde{L}}_{p}\\ \delta {\dot{h}}_{p}={\dot{\tilde{h}}}_{p}-{\dot{h}}_{p}=\delta V_{U_{p}} \end{array}\right.$$

## 4. The Design of Nonlinear Filter Model for Polar Alignment

Aiming at the nonlinear error models derived above, a suitable nonlinear filtering should be selected to replace the standard Kalman filter (KF). Because the extended KF (EKF) has to bear a number of drawbacks such as cumbersome derivation, evaluation of Jacobian matrices, and larger linearized errors and so on [31,32], as a promising substitute for EKF [33], unscented KF (UKF) is employed to accomplish the polar alignment here.

Assuming the measurement errors of accelerometer and gyro are mainly composed of the constant error and zero mean Gaussian white noise. Then we have ${\dot{\varepsilon }}^{b}=0$ and ${\dot{\nabla }}^{b}=0$. Consequently, the filtering state with pseudo frame can be set as:

(53) $$X(t)={\left[\begin{array}{ccccc} {\left(\delta P_{p}\right)}^{T} & {\left(\phi_{p}\right)}^{T} & {\left(\delta V_{p}^{n}\right)}^{T} & {\left(\nabla^{b}\right)}^{T} & {\left(\varepsilon^{b}\right)}^{T} \end{array}\right]}^{T}$$

where $\delta P_{p}$ denotes the pseudo position errors, and $\delta P_{p}={\left[\begin{array}{ccc} \delta L_{p} & \delta \lambda_{p} & \delta h_{p} \end{array}\right]}^{T}$.

According to the nonlinear pseudo error equations in Section 3, the state equation of the filtering model of initial alignment can be obtained as:

(54) $$\left\{ \begin{array}{l} \delta {\dot{P}}_{p}={\left[\begin{array}{ccc} \delta {\dot{L}}_{p} & \delta {\dot{\lambda }}_{p} & \delta {\dot{h}}_{p} \end{array}\right]}^{T}\\ {\dot{\phi }}_{p}=A^{-1}\left[(I-C_{n}^{n^{\prime }}){\tilde{\omega }}_{in}^{n}+C_{n}^{n^{\prime }}\delta \omega_{in}^{n}-C_{b}^{n^{\prime }}\varepsilon^{b}\right]\\ \delta {\dot{V}}_{p}^{n}=\left[I-{\left(C_{n}^{n^{\prime }}\right)}^{T}\right]C_{b}^{n^{\prime }}{\tilde{f}}^{b}-(2\delta \omega_{ip}^{n}+\delta \omega_{pn}^{n})\times {\tilde{V}}_{p}^{n}-(2{\tilde{\omega }}_{ip}^{n}+{\tilde{\omega }}_{pn}^{n})\times \delta V_{p}^{n}+C_{n^{\prime }}^{n}C_{b}^{n^{\prime }}\nabla^{b}\\ {\dot{\nabla }}^{b}=0_{3\times 1}\\ {\dot{\varepsilon }}^{b}=0_{3\times 1} \end{array}\right.$$

where $0_{3\times 1}$ denotes the zero matrix of indicated dimension. 

Further, the general nonlinear system dynamic model can be written by:

(55) $$\dot{X}(t)=f(X,\;t)+W(t)$$

where the specific expressions of f(X, t) can refer to Equation (54), and W(t) is the white system process noise with zero mean and covariance Q(t).

In addition, the differences of the pseudo velocity projected in $g_{p}$-frame between the SINS and external navigation device should be selected as the observations of filter here since the velocity information projected in body frame ($V^{b}$) would be invalid caused by the smaller horizontal component of rotation of Earth. Then, the measurement equation can also be given by:

(56) Z(t)=HX(t)+V(t)

where $H=\left[\begin{array}{ccc} 0_{3\times 6} & I_{3\times 3} & 0_{3\times 6} \end{array}\right]$ is the observation matrix, and V(t) is the white noise with zero mean and covariance R(t).

**Figure 5:**
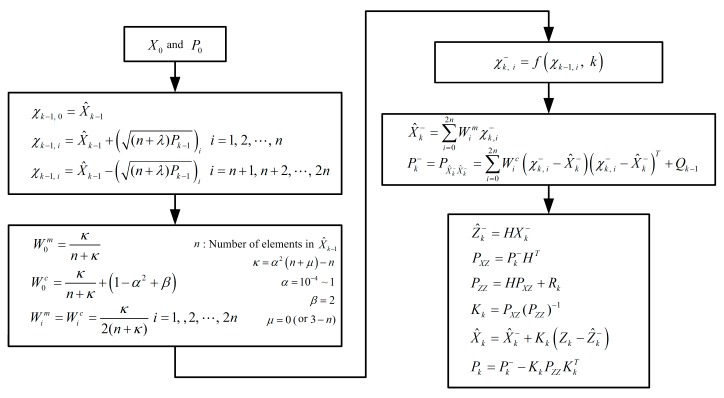
The block diagram of polar alignment with unscented Kalman filter (UKF).

On the other hand, the block diagram of polar alignment assisted by UKF is also shown in Figure 5. For application of UKF to initial alignment with large azimuth misalignment angle, the specific derivation and implement can be found in [30,31,33]. 

## 5. Simulation and Test

In order to confirm the performance of the proposed polar alignment scheme, both semi-physical static simulation and in-motion tests, which are assisted by UKF under the large azimuth misalignment angle, are conducted in this section.

### 5.1. Semi-Physical Static Simulation

Due to the restriction of geography, the semi-physical static simulation is conducted. As attributed to the rotation of Earth, the real static angular velocity measured by gyro at different geographical positions can be exactly known. The angular velocity measured by gyro can be expressed as:

(57) $${\tilde{\omega }}_{ib}^{b}=C_{g}^{b}\omega_{ie}^{g}+\varepsilon^{b}$$

where ${\tilde{\omega }}_{ib}^{b}$ denotes the measured angular velocity; $C_{g}^{b}={\left(C_{b}^{g}\right)}^{T}$ and $C_{b}^{g}$ denotes the attitude matrix of IMU relative to g-frame; $\omega_{ie}^{g}={\left[\begin{array}{ccc} 0 & \Omega \cos L & \Omega \sin L \end{array}\right]}^{T}$, L is the latitude in e-frame.

**Figure 6:**
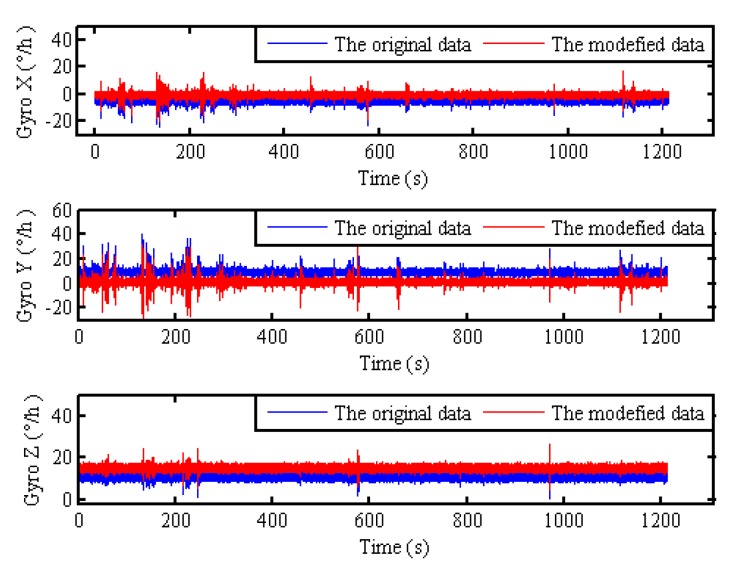
The original outputs of gyro and the modified outputs of gyro with 85° latitude.

Therefore, the static outputs of gyro in polar regions can be simulated by the measured angular velocity in non-polar regions from Equation (57). The static experimental data are collected with fiber optical gyroscope (FOG) SINS, which fixed in a tri-axis high-precision turntable. The attitude reference of IMU is provided by turntable. The gyro drift and the accelerometer bias of test IMU are less 0.01°/h, 100 μg respectively. The local longitude and latitude is 126.6778° and 45.7778°. The pitch, roll and azimuth of IMU relative to the g-frame are 0°, 0° and 30° respectively. The static outputs of IMU for 1200 s are collected, and the update frequency of IMU is 100 Hz. Moreover, according to Equation (57), the original gyro’s outputs and the modified gyro’s outputs with 85° latitude can be shown in Figure 6.

**Figure 7:**
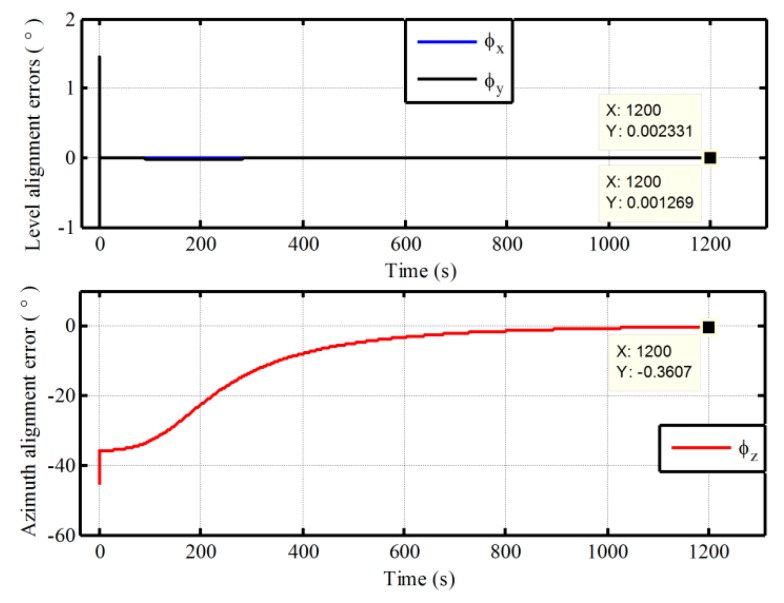
The semi-physical static alignment errors with pseudo navigation mechanization at 85° latitude.

Then, the static alignment with the pseudo-Earth frame is carried out, which is assisted by UKF under the condition of large azimuth misalignment angle. In this simulation, the initial pseudo misalignment angles $\phi_{xp}$, $\phi_{yp}$ and $\phi_{zp}$ are chosen as 1°, 1° and 40°, respectively. Since the position of the vehicle never changes in the static alignment, the strapdown attitude matrix relative g-frame can be obtained directly from Equation (7) without the influence of position errors and the cumbersome computation. As a result, the application of backward process to polar alignment has not been presented here. Through the pseudo attitude matrix and the Equation (7), the static alignment results relative g-frame is obtained directly and is shown in Figure 7.

As shown in Figure 7, the estimated errors of level misalignment angles and azimuth misalignment angle are convergent with time. The level alignment errors can converge rapidly. However, the convergent time of azimuth error is about 1000 s. Because the horizontal component of rotation of Earth decreases to zero gradually with the increase of latitude, the degree of observability of azimuth would become smaller in polar regions. More time is required for azimuth alignment in polar regions. Moreover, it would also be difficult to achieve self-alignment near the pole. Even so, a better alignment result is still obtained with the proposed pseudo navigation mechanization at 85° latitude. The alignment error of azimuth is −0.3607° in 1200 s. Therefore, the proposed pseudo navigation mechanization can solve the problem of traditional attitude reference frame in polar regions caused by SINS mechanization. 

### 5.2. In-Motion Alignment Tests

Due to the restriction of geography, the actual test data of in-motion alignment are difficult to obtain. Consequently, only a vehicle test of low latitude, which is assisted by UKF under the condition of large azimuth misalignment angle, is firstly conducted to verify the feasibility of the proposed alignment scheme. Then, the polar alignment tests with the simulation data are carried out to validate the superior performance of the proposed method. In addition, in the process of polar alignment, the external velocity observation information projected in the navigation frame (namely, $g_{p}$-frame) should be employed to fulfill in-motion alignment tests since the horizontal component of rotation of Earth is very small in and near pole regions.

**Figure 8:**
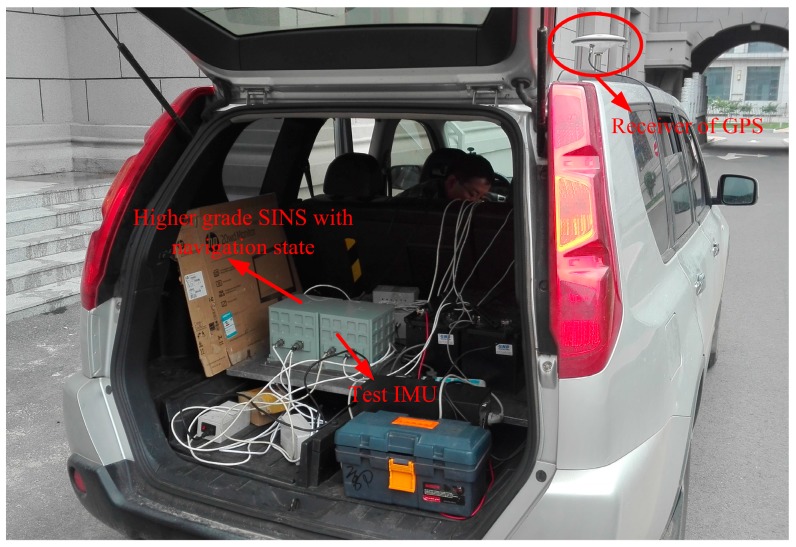
The test vehicle.

**Figure 9:**
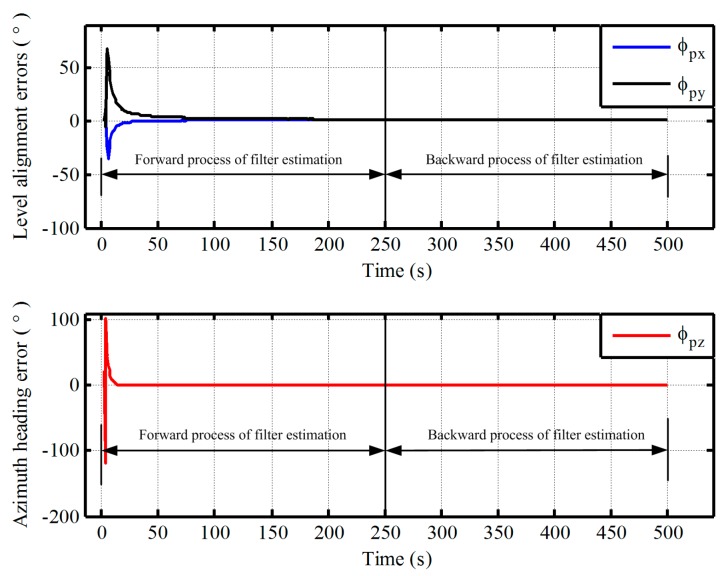
The alignment errors of vehicle test in low latitude regions.

**Figure 10:**
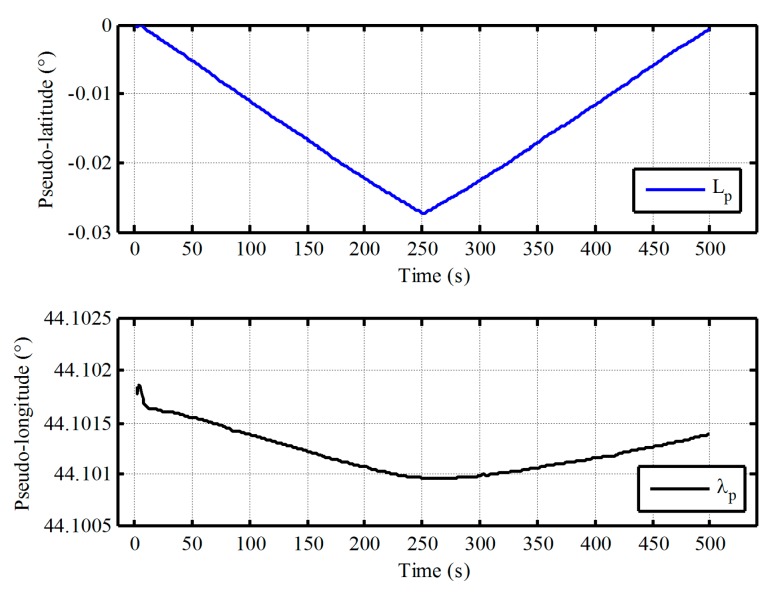
The calculated pseudo position of vehicle test in low latitude regions.

Firstly, the vehicle test of low latitude is executed by equipped with a receiver of differential GPS and two different grades fiber optical gyroscope (FOG) SINSs as shown in Figure 8. The gyro constant drift and the accelerometer constant bias of test IMU are less 0.01°/h, 100 μg respectively. The local longitude and latitude are 126.6778° and 45.7778°, respectively. In total, 250 s vehicle data were collected. The initial pseudo misalignment angles $\phi_{xp}$, $\phi_{yp}$ and $\phi_{zp}$ are chosen as 1°, 1° and 20°, respectively. In addition, since this paper mainly focuses on the design of polar alignment scheme, the application of backward process to accelerate the alignment process more has not been presented. Only a backward process is conducted to implement polar alignment and accelerate the alignment process here. For the application of backward process to decrease the alignment time further, more details can be found in [27,28,29]. Here, the filtering estimation of initial alignment firstly works with test data by the forward process. Subsequently, the filtering estimation is carried out with the opposite time sequence. The alignment errors and the calculated pseudo position are shown in Figure 9 and Figure 10, respectively.

As shown in Figure 9, the estimated errors of three-axis misalignment angles are convergent with time. The pseudo position can also return to the initial position with the backward process when the initial alignment is completed from Figure 10. Although there are still some differences between the initial value and the final value of pseudo position, and the drift errors of pseudo position are unavoidable because the external velocity information is selected as the observation information of filter in the process of initial alignment, the influence of the drift errors of pseudo position on the initial transformation matrix is very small and can be neglected since the initial pseudo latitude is always zero. Therefore, the pseudo strapdown attitude matrix of vehicle at initial position is obtained at the end of alignment phase. Then, the initial attitude matrix relative to g-frame can be obtained directly with Equation (7). There is less cumbersome derivation and low computational complexity in the process of transformation since the initial transformation matrix is a fixed known constant matrix. Consequently, we can conclude that the proposed polar alignment scheme would be feasible for in-motion alignment and has less complexity.

**Figure 11:**
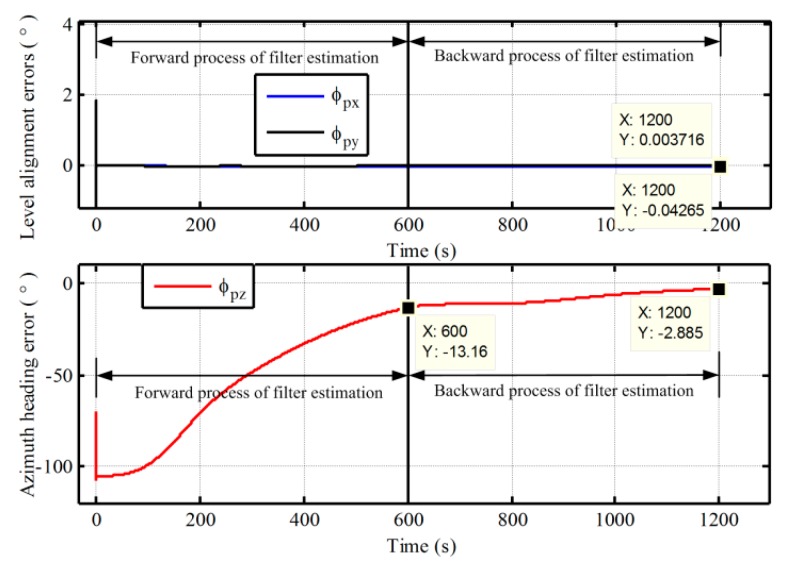
The in-motion alignment errors based on the proposed polar alignment algorithm with initial latitude 87°.

**Figure 12:**
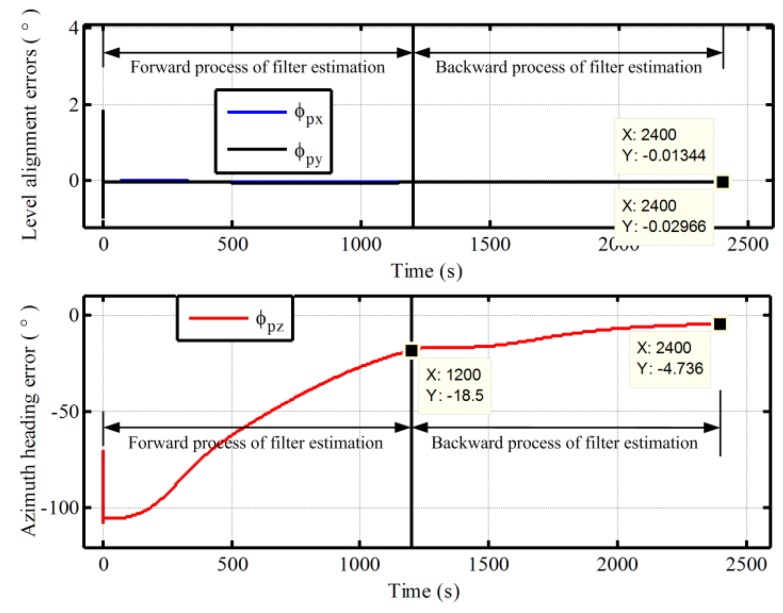
The in-motion alignment errors based on the proposed polar alignment algorithm with initial latitude 90°.

**Figure 13:**
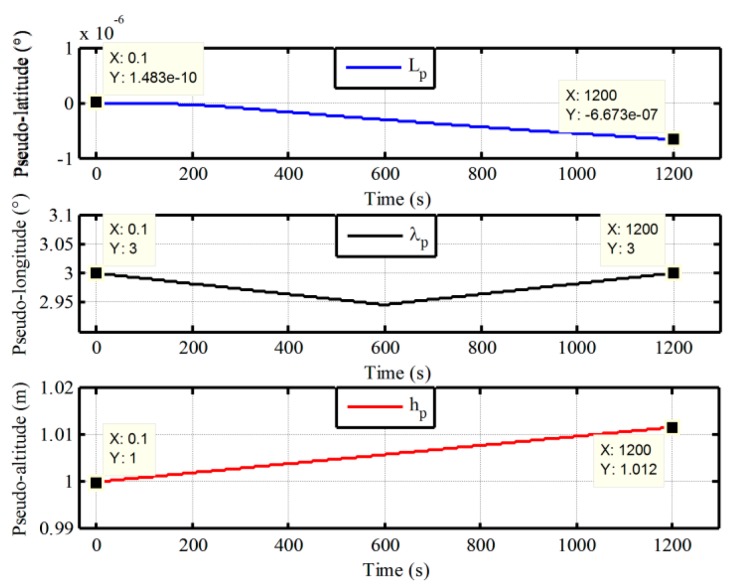
The calculated pseudo-position with initial latitude 87°.

**Figure 14:**
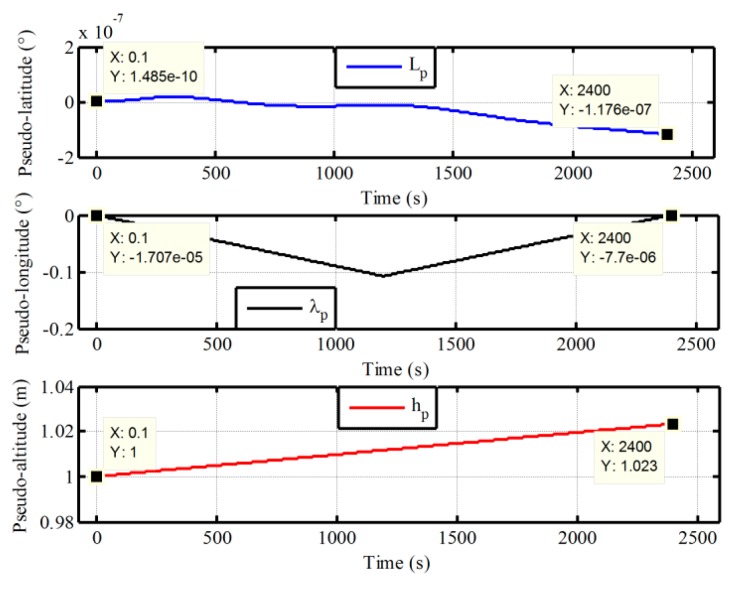
The calculated pseudo-position with initial latitude 90°.

Next, the superior performance of the proposed method for polar alignment is verified by simulation data due to restriction of geography. In polar in-motion alignment simulations, the outputs of gyro and accelerometer are generated by a strapdown INS simulator. The constant and random drifts of gyro are 0.01 °/h and 0.001 °/h, respectively, and the constant and random biases of the accelerometer are 100 μg and 10 μg, respectively. The initial values of attitude relative to g-frame are all set to be zero, and a constant velocity of 10m/s in the along-vehicle direction is applied all the scenarios. According the definitions of pseudo-Earth frame, the vehicle would sail along the pseudo equator with the constant velocity of 10 m/s. Two groups of in-motion data with the different initial latitudes 87° and 90° are obtained. The initial longitudes are all selected as 126°. The length of test data with the initial latitude 87° is 600 s, while the length of test data with the initial latitude 90° is 1200 s. The update frequency of IMU data is 100 Hz. In the process of initial alignment, the initial pseudo position errors $L_{p}$,$\lambda_{p}$, and $h_{p}$ are all set to be 1 m. The initial pseudo velocity errors are all 0.1 m/s. The initial pseudo misalignment angles $\phi_{xp}$, $\phi_{yp}$, and $\phi_{zp}$ are chosen as 1°, 1°, and 70°, respectively. The alignment results of 87° latitude and 90° latitude are shown in Figure 11 and Figure 12, respectively. The calculated pseudo positions with the pseudo navigation mechanization are also shown in Figure 13 and Figure 14. In addition, a comparison with the traditional method based on transverse frame at 87° latitude is also carried out. The final alignment result relative to g-frame based transverse frame is obtained by direct transformation with the calculated transverse position from transverse SINS calculation. The mean and variance of 30 alignment errors in 1200 s are shown in Table 1. 

As shown in Figure 11 and Figure 12, at the end of the first forward process, the azimuth alignment errors of 87° and 90° are −13.16° in 600 s and −18.5° in 1200 s, respectively. The alignment errors of azimuth are still larger. While the alignment errors of azimuth are −2.885° and −4.736°, respectively, at the end of the backward process. As a result, the proposed alignment scheme with the backward process extends the length of dada equivalently and can utilize the data adequately to decrease the alignment time. Moreover, the alignment performance of the proposed algorithm is also very favorable. The alignment accuracy can meet the requirement of polar alignment. Therefore, the proposed alignment algorithm can solve the problem of polar alignment caused by SINS mechanization.

On the other hand, in Figure 13 and Figure 14, it is clear that the pseudo position returns to the initial position, when the initial alignment is completed. Although there are still some differences, the influence of the drift errors of pseudo position on the initial transformation matrix $C_{g}^{g_{p}}(0)$ is very small and can be neglected since the initial pseudo latitude is always zero. Therefore, the initial transformation matrix $C_{g}^{g_{p}}(0)$ can be utilized to obtain the strapdown attitude matrix with a simple transformation logic and without influence of position errors. Moreover, with the simple transformation logic, there have less the cumbersome derivation and low computational complexity in the process of transformation, thereby decreasing the complexity of program and the burden of computer. Here, we should notice that the azimuth angle relative to g-frame is the angle between the vehicle direction and the 126° longitude when the initial latitude is 90°. In addition, in Table 1, we can see that the mean and variance of alignment errors based on the proposed algorithm are also smaller as compared with the traditional method. This is because the proposed algorithm eliminates the influence of position errors on transformational accuracy. Therefore, the proposed algorithm has superior performance. On the other hand, since the strapdown attitude matrix relative to g-frame is obtained at the end of alignment phase, it would also be more convenient to access any one of the following polar navigation systems without any additional workloads and complexities. Because the existing transformation program of navigation parameters in global navigation system can be utilized directly, the proposed polar alignment algorithm would be advantageous to unify the forms of polar alignment and the external observation information of filter. 

Furthermore, comparing Figure 11 and Figure 12, more time is required for polar alignment with the initial latitude 90°. This is the other key problem, which must be confronted in polar alignment. The degree of observability of azimuth is smaller in polar regions since the horizontal component of rotation of Earth decreases to zero gradually with the increase of latitude. As a result, the future efforts will focus on the improvement of the degree of observability of azimuth, thereby improving the polar alignment performance more.

## 6. Conclusions

In this paper, from the perspective of global inertial navigation, a generalized transverse Earth frame built based on the initial position of vehicle (namely, pseudo-Earth frame) is proposed as the attitude reference frame for polar alignment. Then, a polar alignment algorithm based on the pseudo-Earth frame and backward process is proposed to implement polar alignment. Through the proposed algorithm, the attitude matrix relative to local geographic frame is obtained without influence of position errors when the initial alignment is completed, and there is less cumbersome derivation and low computational complexity. As a result, it is very convenient to access any one of the following polar navigation systems without any additional workloads and complexities. Moreover, it would also be advantageous to unify the forms of polar alignment and the external observation information of filter, thereby further unifying the implemented form of the reference information acquired from the external navigation device. In addition, the backward process can still accelerate the filtering estimation and decrease the alignment time. Finally, the semi-physical static simulation and in-motion tests with large azimuth misalignment angle assisted by UKF are carried out to verify the proposed polar alignment algorithm, and the results exhibit the superior performance of the proposed alignment algorithm in polar regions.

## Figures and Tables

**Table 1:** Statistics of alignment errors based on the two methods at 87° latitude in 1200 s.

Errors	The Proposed Method		Traditional Transverse Method	
	Mean	Variance	Mean	Variance
Pitch	0.003108°	5 × 10^−7^	0.005417°	4.25 × 10^−5^
Roll	−0.04375°	7.63 × 10^−4^	0.07080°	2.6 × 10^−4^
Azimuth	−2.869°	3.26 × 10^−4^	−3.127°	8.64 × 10^−3^
